# High Degree Atrioventricular Block in Non-ST-Segment Elevation Myocardial Infarction (NSTEMI) and Role of Early Revascularization

**DOI:** 10.7759/cureus.9222

**Published:** 2020-07-16

**Authors:** Raj Patel, Adrian Krukowski, Amjad Sheikh

**Affiliations:** 1 Internal Medicine, Weiss Memorial Hospital, Chicago, USA; 2 Internal Medicine, Louis A Weiss Memorial Hospital, Chicago, USA; 3 Interventional Cardiology, Louis A Weiss Memorial Hospital, Chicago, USA

**Keywords:** nstemi, high degree av block, dual atrioventricular nodal supply, revascularization, pacemaker’s implantation, cardiac conduction disorder

## Abstract

High degree atrioventricular block (HDAVB) is a rare complication of non-ST-elevation myocardial infarction (NSTEMI) and often requires pacemaker implantation. We describe a case of reversal of HDAVB in a patient with dual atrioventricular node blood supply following coronary revascularization and potential mechanisms of such complications.

## Introduction

As the prevalence of coronary artery disease in the general population increases, so does the incidence of non-ST-segment elevation myocardial infarction (NSTEMI) [1]. An uncommon life-threatening complication of NSTEMI is the development of a high degree AV block (HDAVB), where the atria and ventricles become almost completely dissociated. While the mechanisms of such complications are not well understood, only a few cases demonstrate the reversal of HDAVB following coronary revascularization. Here, we report a case of HDAVB reversal in NSTEMI following coronary revascularization in a patient with a co-dominant coronary artery circulation.

## Case presentation

**Figure 1:**
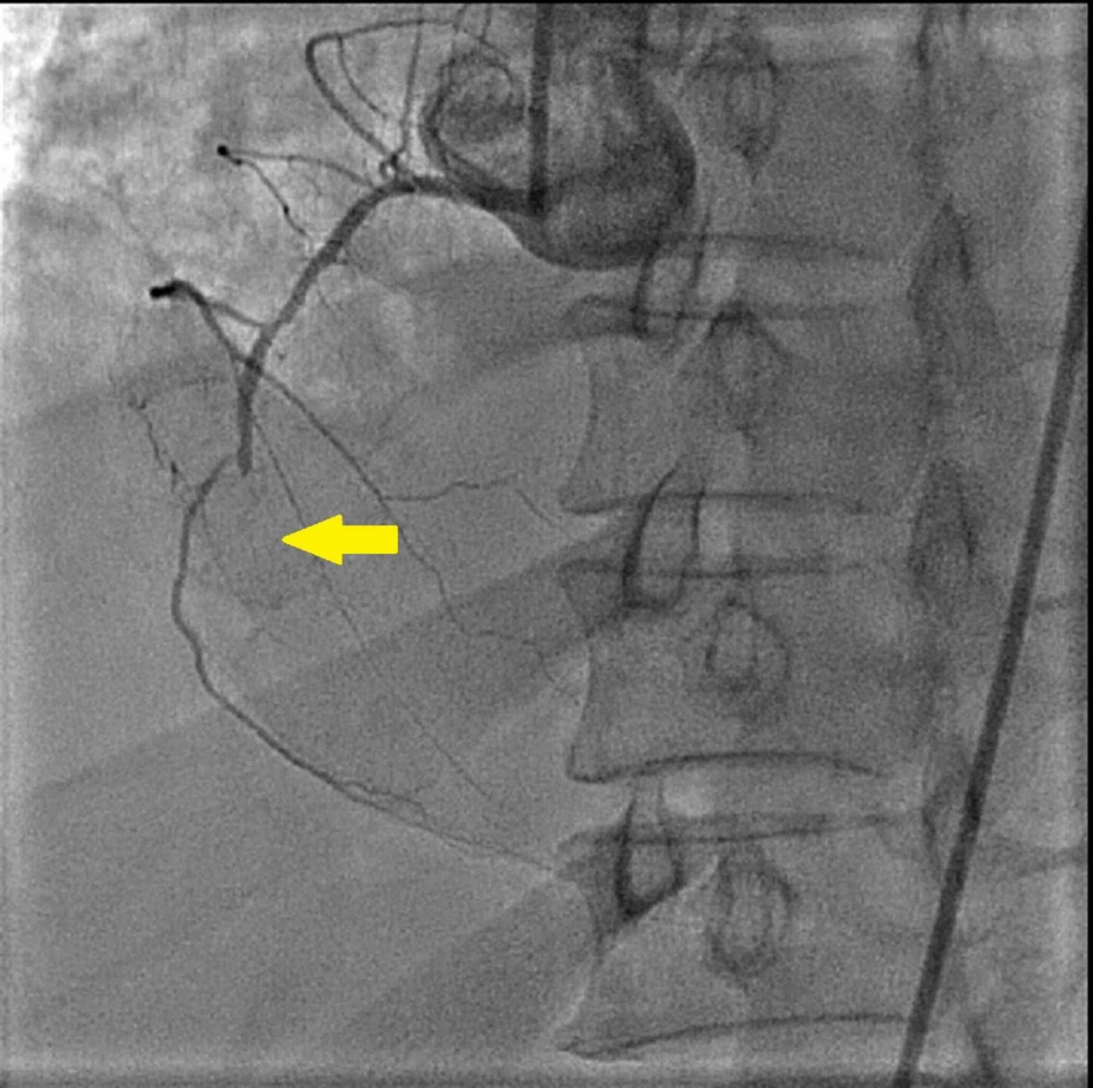
Totally occluded right coronary artery can be seen in the mid portion after giving rise to right ventricular branch

**Figure 2:**
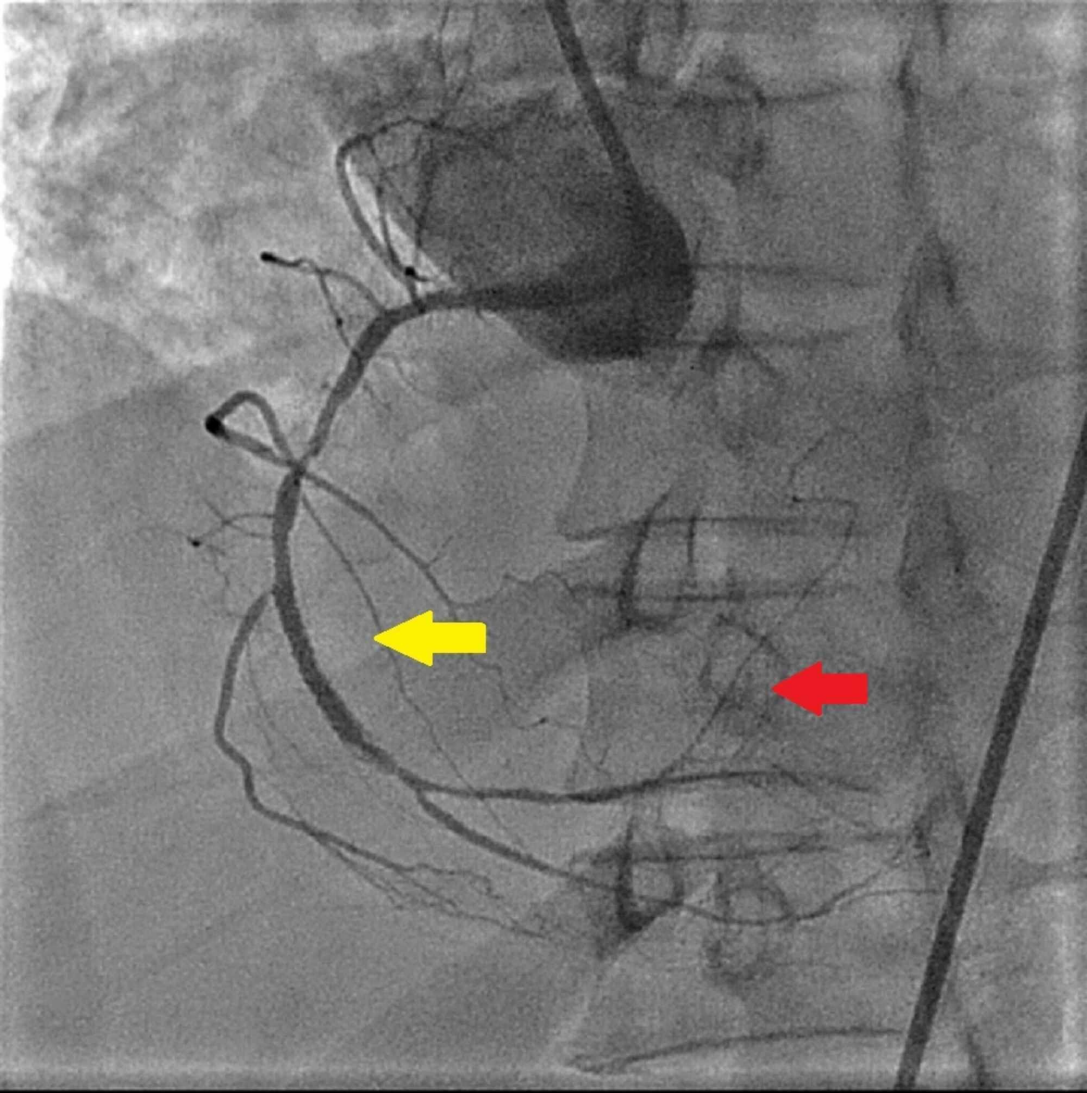
Successful drug-eluting stent placement in the right coronary artery (yellow arrow) and visualization of AV nodal artery (red arrow)

**Figure 3:**
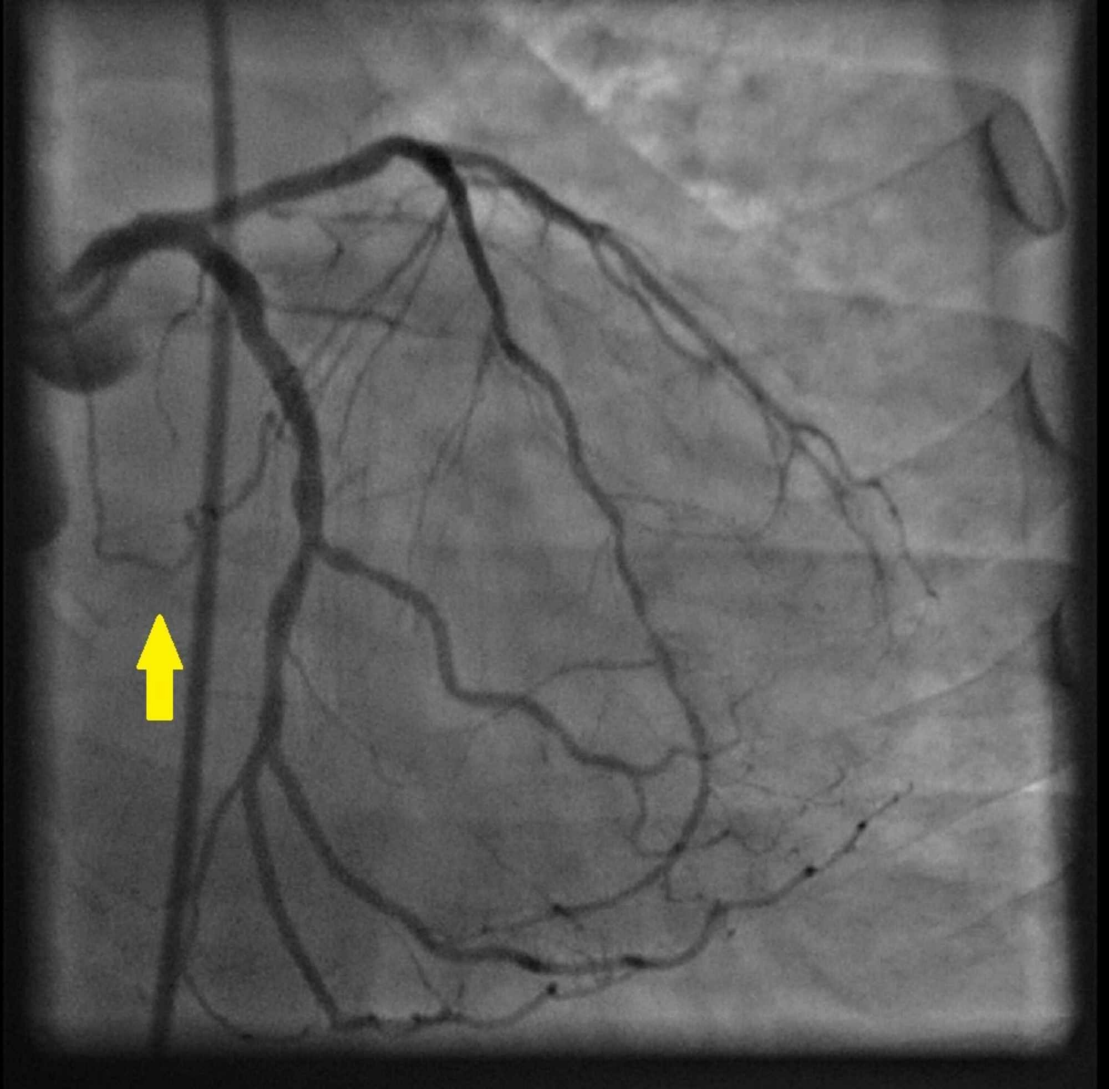
Atrioventricular circumflex artery originating from left circumflex artery

**Figure 4:**
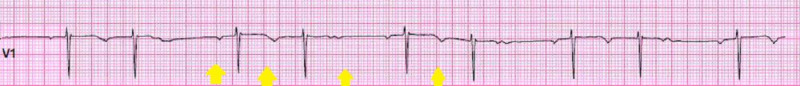
Sinus rhythm with atrioventricular dissociation and junctional bradycardia before revascularization

**Figure 5:**
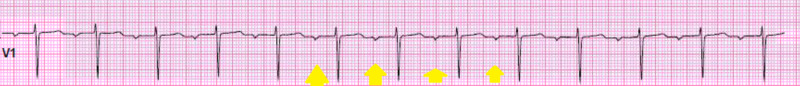
Sinus rhythm with 1st degree AV block after revascularization

A 47-year-old female, with a past medical history of hypothyroidism and no significant cardiac history, was admitted with complaints of atypical chest pain and generalized weakness. An initial electrocardiogram (EKG) was performed that revealed 1st-degree atrioventricular (AV) block with a ventricular rate of 42 beats per minute. Physical examination was unremarkable, and labs were significant for a Troponin-I level of 4.89 ng/ml. Emergent cardiac catheterization revealed complete occlusion of the right coronary artery (RCA) (Figure 1), and a drug-eluting stent was successfully deployed in the distal RCA (Figure 2). Minimal coronary disease of the left main and left circumflex artery was noted, and a patent AV circumflex branch originating from the left circumflex artery (LCA) was visualized (Figure 3). Two hours following initial treatment with aspirin and therapeutic enoxaparin, a repeat EKG showed conduction block progression to high degree AV block (Figure 4). However, following revascularization an additional AV nodal artery was also visualized originating from the RCA, establishing co-dominant coronary circulation. Following revascularization, the patient's conduction abnormalities gradually resolved and returned to 1:1 AV conduction (Figure 5), thus avoiding the need for cardiac pacemaker placement.

## Discussion

The resolution of high degree AV block following RCA revascularization has been previously described, but this is the first case of HDAVB resolution in the presence of co-dominant coronary circulation in the setting of NSTEMI [2].

HDAVB is a form of advanced second-degree heart block. It is differentiated from complete heart block by the conduction of some atrial impulses to the ventricles and absence of complete AV dissociation, whereas in former, there is no conduction of atrial impulses and there is complete AV dissociation. In an analysis of nationwide inpatient database, 0.6% of patients admitted with NSTEMI developed HDAVB, and a third of those patients required permanent pacemaker placement [3]. Overall, rates of post-MI conduction abnormalities are decreasing given increasing primary percutaneous coronary intervention (PCI). Most studies have reported 1 to 3 percent of patients experiencing such abnormalities [4]. However, HDAVB is associated with higher mortality because of a greater extent of myocardial and conduction system involvement. In NSTEMI, there are two possible mechanisms previously described for such complications: (1) According to vagal theory, the infarcted area causes stretch and distension of myocardium and release of chemical substances (prostaglandins, serotonin, free radicals) which activate efferent vagal fibers. This leads to hypotension and bradyarrhythmia. This phenomenon is also known as Bezold-Jarisch reflex [5]; (2) According to ischemia theory, AV conduction defects are directly related to ischemia with RCA occlusion, and such defects resolve with revascularization, as seen in our patient.

In our case, the patient presented with 1st degree AV block which quickly progressed to HDAVB and the patient required emergent percutaneous intervention. Left heart catheterization demonstrated variant coronary anatomy seen in only 2% of patients, where the AV nodal branch originates from both the RCA and LCA [6]. The return of 1:1 conduction following revascularization supports ischemia theory and ultimately averted the need for pacemaker placement and associated complications. Pacemaker placement is recommended in patients with symptomatic second- or third-degree AV block and considering pacemaker in asymptomatic patients [7]. ACC/AHA guidelines recommend management of HDAVB with ischemia-guided strategy, but there is no data available regarding whether the timing of the revascularization is associated with reversibility of such conduction abnormalities and remains to be studied [8].

## Conclusions

We report first case of the patient with dual atrioventricular nodal supply presenting with NSTEMI complicated by high degree AV block. Rapid identification and early coronary revascularization of this abnormality may lead to reversal of conduction block and establishing 1:1 AV conduction thus avoiding the need for pacemaker insertion. Our case supports the ischemia-related mechanism of such complications and emphasizes the role of early intervention in order to improve outcomes.
